# Day-to-day fluctuation of point-of-care circulating cathodic antigen test scores and faecal egg counts in children infected with *Schistosoma mansoni* in Ethiopia

**DOI:** 10.1186/1471-2334-14-210

**Published:** 2014-04-17

**Authors:** Abraham Degarege, Mengistu Legesse, Girmay Medhin, Tilahun Teklehaymanot, Berhanu Erko

**Affiliations:** 1 Aklilu Lemma Institute of Pathobiology, Addis Ababa University, Addis Ababa, Ethiopia

**Keywords:** *Schistosoma mansoni*, Urine-CCA cassette, Kato-Katz method, Variation, Ethiopia

## Abstract

**Background:**

Determining the variation of circulating cathodic antigen (CCA) in urine and egg counts variation in stool between days in *Schistosoma mansoni* (*S. mansoni*) infected individuals is vital to decide whether or not to rely on a single-sample test for diagnosis of Schistosomiasis. In this study, the magnitude of day-to-day variation in urine-CCA test scores and in faecal egg counts was evaluated in school children in Ethiopia.

**Methods:**

A total of 620 school children (age 8 to 12 years) were examined for *S. mansoni* infection using double Kato-Katz and single urine-CCA cassette methods (batch 32727) on three consecutive days.

**Results:**

The prevalence of *S. mansoni* infection was 81.1% based on triple urine-CCA-cassette test and 53.1% based on six Kato-Katz thick smears. Among the study participants, 26.3% showed fluctuation in urine CCA and 32.4% showed fluctuation in egg output. Mean egg count as well as number of cases in each class of intensity and intensity of cassette band color varied over the three days of examination. Over 85% of the children that showed day-to-day variations in status of *S. mansoni* infection from negative to positive or vice versa by the Kato-Katz and the CCA methods had light intensity of infection. The fluctuation in both the CCA test scores and faecal egg count was not associated with age and sex.

**Conclusions:**

The current study showed day-to-day variation in CCA and Kato-Katz test results of children infected with *S. mansoni*. This indicates the necessity of more than one urine or stool samples to be collected on different days for more reliable diagnosis of *S. mansoni* infection in low endemic areas.

## Background

*S. mansoni* infection is a common public health problem in the developing world [1]. It affects about 243 million people living in the sub-Saharan Africa [2]. Infection is particularly prevalent in children causing serious problems such as cognitive impairment, undernourishment and retarded growth [3,4].

Effective control of *S. mansoni* infection needs accurate diagnosis and treatment of cases [5]. Kato-Katz is the method advocated for diagnosis of *S. mansoni* infection for field epidemiological survey [6]. However, its low sensitivity in light infection and variability in eggs released in stool limit its use in low endemic areas for *S. mansoni*[7,8]. As a result, the urine-CCA method has been in field trial for several years to be used as an alternative tool for the diagnosis of *S. mansoni* infection [9-12]. Currently, the urine-CCA cassette (batch 32727) has been recommended for mapping *S. mansoni* infection [13]. This specific urine-CCA cassette was found to be better in its performance than the Kato-Katz method for the diagnosis of *S. mansoni* infection [13,14]. On the other hand, day-to-day variation in CCA excretions in urine [11,12,15-18] is one of the limitation in relying on this method for the effective diagnosis of cases. Thus, it is important to study the antigen variation in urine while applying the test for the diagnosis of *S. mansoni* infection in order to decide on the possibility of relying on a single-sample test. Moreover, the effect of geographical variations of *S. mansoni* strains on the day-to-day variability of CCA antigens released in urine is poorly understood. Therefore, the objective of this study was to evaluate day-to-day variation in urine-CCA test scores and in faecal egg counts while using the urine-CCA cassette test (batch 32727) and the Kato-Katz thick smear for the diagnosis of *S. mansoni* infection in children. The current paper is building on the same data set reported in Erko et al. [14], but different analysis was used to approach day-to-day variation in urine-CCA cassette test and egg out puts in the diagnosis of *S. mansoni* infection.

## Methods

### Study area and population

A cross-sectional study involving 620 children (320 from Jiga and 300 from Harbu Elementary Schools located in Jiga and Harbu towns; age ranged: from 8 to12 years and 51% were females) was conducted in 2010 and 2011. Details of the study sites and study population have been described in Erko et al. [14].

### Urine specimen collection and testing

Midstream urine specimens were collected for three consecutive days from each study participant. The urine POC/CCA (point-of-care circulating cathodic antigen) cassette test was performed according to the protocol and procedures described by the manufacturer (http://www.rapid-diagnostics.com/downloads/RMD%20Pamphlet%202011_06_13%20.pdf). The urine samples were tested with POC-CCA immediately after collection, and the reading was carried out by one laboratory technician about 20 minutes after the buffer was added to the sample. Any line in the test area was considered positive and the results were qualitatively recorded as a strong and weak based on the intensity of the band.

### Stool collection and examination

On the same days about 2 gram of stool sample was collected from each of the study participants who provided urine specimens. Two slides were prepared per sample using a 41.7 mg Kato-Katz template [19] and quantitatively examined for *S. mansoni* by two laboratory technicians 24 hours after smear preparation. The third technician checked the reading of the two technicians and in case of discrepancies the slides were re-examined. There was an agreement (>95%) between technicians in reading the Kato Katz slides. The results were recorded as egg count per slide and multiplied by 24 to convert it into eggs per gram (epg) of stool for data analysis.

### Ethical considerations

The study was ethically approved by the Institutional Review Board (IRB) of the Aklilu Lemma Institute of Pathobiology, Addis Ababa University. Permission to conduct the study was also obtained from local government administration and school directors. The children were included in the study after obtaining informed consent from their parents/guardians. In addition, the objective of the study was clearly explained to the children and their assent was sought. Children found positive for intestinal schistosomiasis and soil-transmitted helminthiasis were treated with a single dose of Praziquantel (40 mg/kg body weight) and Albendazole (400 mg), respectively after the third day of specimen collection and examination.

### Data analysis

Data were double entered and cross-checked using Excel 2007 sheet and analyzed using STATA (version 10). Pearson chi-squared test was used to test difference in proportions of fluctuations between sex, age, location and infection intensity groups. McNemar's chi-squared test was used to compare prevalence of *S. mansoni* infection between days based on the Kato-Katz as well as the urine-CCA cassette test. Analysis of variance for repeated measures was used to compare differences in mean egg counts among the three days. The agreement in the status of *S. mansoni* infection between the days was evaluated using Cohen's kappa coefficient. Cohen's kappa values < 0 were interpreted as indicating no agreement and 0–0.20 as slight, 0.21–0.40 as fair, 0.41–0.60 as moderate, 0.61–0.80 as substantial, and 0.81–1 as almost perfect agreement on the status of *S. mansonia* infection of the study participants between two different days [20]. The arithmetic mean egg count was calculated as the average egg counts (epg) of 6 Kato-Katz thick smears and classes of intensity of *S. mansoni* infection was determined based on this result as light (1–99 EPG), moderate (100–399 EPG) and heavy (≥400 EPG). 95% confidence interval (CI) was estimated for each diagnostic performance parameter. Results were considered significant when *p* < 0.05.

## Results

### *S. mansoni* infection status based on the urine-CCA cassette test

Using urine-CCA cassette test, 54.8% (340/620) of the children were positive for *S. mansoni* infection on all the three consecutive days, 18.9% (117/620) were negative in all the three consecutive days, and 26.3% (163/620) had fluctuating results (at least two days results are different). Out of 441 children who had positive test results on day 1, 13.6% were negative on day 2, 16.8% were negative on day 3 and 8.3% were negative both on day 2 and day 3. Out of 179 children whose day 1 urine-CCA test result was negative, 24.6% were positive on day 2, 20.7% were positive on day 3 and 10.1% were positive both on day 2 and day 3. Sixty five children who were positive for *S. mansoni* on day 2, were negative on day 3 and 43 children who were negative for *S. mansoni* on day 2, were positive on day 3 (Table 1). Twenty six children (4.2%) were positive for *S. mansoni* on day 2 but negative on both day 1 and day 3. Similarly, 19 children (3.1%) were positive for *S. mansoni* on day 3, but negative on both day 1 and day 2. There was significant variability in the positivity/negativity and band color intensity of the urine-CCA cassette test for *S. mansoni* infection between day 1 and day 2, day 1 and day 3, as well as day 2 and day 3 (Table 1). The fluctuation in test results of the POC-CCA test was more frequent among children who showed weak intensity band color. Variation in urine-CCA cassette test over the three days of examination decreased as the intensity of infection increased. The urine-CCA cassette test showed less variation compared to Kato-Katz over the three days of examination.

**Table 1:** **Day-to-day variations in status of children in Tikur Wuha and Harbu elementary schools for *S. mansoni *** **infection as determined using single urine-CCA cassette test, Ethiopia**

	**Day 3**				**Day 2**		
	**Negative**	**Weak positive**	**Strong positive**	**Total**	**Negative**	**Weak positive**	**Strong positive**
**Day1**							
Negative	142	22	15	179	135	35	9
Weak positive	49	35	32	116	41	42	33
Strong positive	25	36	264	325	19	40	266
Total	216	93	311	620	195	116	308
Kappa	0.53				0.53		
**Day 2**							
Negative	152	25	18				
Weak positive	44	33	39				
Strong positive	21	33	254				
Kappa	0.53						

Prevalence of *S. mansoni* infection based on cumulative CCA-results of day 1 and day 2 examinations (78.2%) as well as cumulative results of day 1 and day 3 (77.1%) were significantly higher than the prevalence of *S. mansoni* infection determined by CCA results on day 1 (71.1%), day 2 (68.5%) and day 3 (65.2%) (p < 0.05 for all). Prevalence of *S. mansoni* infection based on cumulative CCA-results of day 2 and day 3 examination (75.5%) was also significantly higher than the prevalence determined by CCA results of day 2 (68.5%) or day 3 (65.2%) examination (p < 0.05 for both). Similarly, prevalence of *S. mansoni* based on cumulative CCA-results of the three consecutive days examination (81.1%) was significantly higher than the prevalence determined based on cumulative results of day 2 and day 3 (75.3%) (p = 0.01) examination. However, differences in prevalence of *S. mansoni* based on cumulative CCA-results of the three days examination and day 1 and day 2 (78.2%) or day 1 and day 3 (77.1%) was not significant.

### *S. mansoni* infection status based on the Kato-Katz thick smear method

There was moderate agreement of *S. mansoni* infection when results were compared between day 1 and day 2 (Kappa = 0.42), between day 1 and day 3 (Kappa = 0.45), and between day 2 and day 3 (Kappa = 0.46) (Table 2). Among the participants, 32.4% (201/620) showed variation in Kato-Katz results over the three days of examination. However, 25.5% (158/620) remained positive and 42.1% (261/620) remained negative over the three days of examination using the Kato-Katz method. Out of 251 children who were diagnosed positive for *S. mansoni* infection on day 1, 170 (67.7%) were positive on day 2 and 182 (72.5%) were positive on day 3. Similarly, among 369 children who were negative for *S. mansoni* infection on day 1, 311 (84.3%) remained negative on day 2 and 315 (85.4%) remained negative on day 3. Fifty nine children who were positive for *S. mansoni* on day 2 were found negative on day 3 and 75 children who were negative for *S. mansoni* on day 2 were positive on day 3. Among the study participants who were positive for *S. mansoni* infection on day 1, 17.6% (44/251) were negative on both day 2 and day 3, and 13.3% (30/225) of the participants who were positive for *S. mansoni* infection on day 2 were negative on both day 1 and day 3.

**Table 2:** **Day-to-day variations in status of children in Tikur Wuha and Harbu elementary schools for *S. mansoni *** **infection when determined using the Kato-Katz method, Ethiopia**

	**Day 3**					**Day 2**			
	**Negative**	**Light**	**Moderate**	**Heavy**	**Total**	**Negative**	**Light**	**Moderate**	**Heavy**
**Day1**									
Negative	311	50	7	1	369	315	49	3	2
Light	60	85	21	6	172	69	77	22	3
Moderate	7	21	30	6	64	8	21	30	5
Heavy	2	2	4	7	15	3	2	2	8
Total	380	158	62	20	620	395	149	57	18
Kappa	0.45					0.42			
**Day 2**									
Negative	320	60	13	2					
Light	55	78	16	1					
Moderate	4	17	30	6					
Heavy	1	3	3	11					
Kappa	0.46								

Majority of the participants (>85%) who were negative for *S. mansoni* infection on day 1, but positive on day 2 or day 3, and positive on day 1, but negative on day 2 or day 3 had light intensity of infection. Among children who were positive for *S. mansoni* infection, there was fluctuation in the intensity of infection among the three days of examination. The difference in mean egg count of *S. mansoni* observed on day 1 (mean egg per gram (mepg) = 133.4, 95% CI = 106.0, 160.9), on day 2 (mepg = 148.4, 95% CI = 115.5, 181.4) and on day 3 (mepg = 150.6, 95% CI = 122.6, 178.6) were not significant. Majority of the children (>85%) that showed day-to-day variations in the status of *S. mansoni* infection from negative to positive or vice versa by Kato-Katz as well as by the CCA methods had light intensity of infection.

### Factors associated with day-to-day variation in CCA and faecal egg counts

Larger proportion of individuals who had light intensity of infection (70.7%) showed fluctuation in *S. mansoni* infection status over the three days of examination compared to those with moderate (17.8%) or heavy intensity (11.8%) of infection when examined using duplicate Kato-Katz and the urine-CCA method (p < 0.01) (Table 3). Similarly, the percentage of individuals who showed fluctuation in *S mansoni* infection status over the three days of examination was higher in those from Harbu area (39.3%) than those from Jiga (25.9%) area when examined using duplicate Kato-Katz method. However, the percentage of individuals who showed fluctuation in *S mansoni* infection status over the three days of examination was comparable between males and females, and between individuals of age 5 to 9 and 10 to 15 years when examined by the duplicate Kato-Katz as well as the urine-CCA methods.

**Table 3:** **Day-to-day variations in status of children for *S. mansoni *** **infection based on age groups, sexes, location and intensity of infection as determined using single urine-CCA cassette test and duplicate Kato-Katz method**

**Variable**	**Number examined**	**Number fluctuated over the three days of examination based on Kato-Katz (%) method**	**Number fluctuated over the three days of examination based on the urine-CCA (%) test**
**Age group**			
5-9	237	85 (35.9)	65 (27.4)
10-12	383	116 (30.3)	97 (25.30)
Total	620	201 (32.4)	162 (26.1)
X^2^ (*p*)		3.4 (0.180)	0.39 (0.531)
**Sex**			
Female	316	101 (31.9)	86 (27.5)
Male	304	100 (32.9)	76 (25.0)
X^2^(*p)*		0.03 (0.865)	0.52 (0.471)
**Location**			
Jiga	320	83 (25.9)	90 (28.1)
Harbu	300	118 (39.3)	72 (23.8)
X^2^(*p*)		13.23 (<0.01)	1.85 (0.174)
**Intensity of infection**			
Light	239	169 (70.7)	40 (20.1)
Moderate	73	13 (17.8)	5 (6.8)
Heavy	17	2 (11.8)	0 (0.0)
X^2^ (*p*)		85.35 (<0.01)	11.45 (0.003)

## Discussion

In the current study, fairly large number of study participants showed changes in their status of *S. mansoni* infection when examined using the Kato-Katz and urine-CCA methods over three consecutive days. Prevalence of *S. mansoni* infection increased significantly when results were compared between a single day and cumulative of two or three days.

Out of the 503 *S. mansoni* positive participants based on the cumulative results of the three days examination using a single urine-CCA cassette test, only 340 were found consistently positive for the parasite in all the three days. Among 280 children who were negative for *S. mansoni* infection at least on one of the three days of examination using a single urine-CCA cassette test, only 117 children were found consistently negative for the parasite during the three days of examination using the test. About 26.3% of the participants showed changes in the status of *S. mansoni* infection using the urine-CCA cassette test when compared among the three days of examination. Consequently, prevalence of *S. mansoni* infection among the participants increased when the result was analyzed based on examination done on day 1, day 2 or day 3 and cumulative of day 1 and day 2, day 1 and day 3, day 2 and day 3, and day1, day 2 and day 3. Previous studies also showed inconsistency in positivity and negativity of individuals for *S. mansoni* infection when examined using the urine-CCA cassette method in different days [11,12,15]. One possible explanation for this variability of CCA test results could be attributed to the day-to-day fluctuation of *S. mansoni* CCA levels in urine as previously reported [16-18].

Some participants who were tested positive were found negative or vice versa when examined over the three days using Kato-Katz method. Fairly large number of individuals who were positive for the parasite egg on one of the three days were found negative on the other two days of examination. As a result, prevalence of *S. mansoni* infection was higher based on samples collected on 3 days (6 Kato-Katz slides) than on two (4 Kato-Katz slides) or single day (2 Kato-Katz slide). This agrees with the previous observation of day-to-day variation in status of individuals for *S. mansoni* infection as determined using the Kato-Katz method [7,8]. Had larger samples from several schools were taken, the prevalence/intensity of infection for the areas might be estimated based on results of only one Kato-Kato slide on each day. This would be helpful in evaluating the nature of the treatment that can be recommended for the areas.

The fluctuations observed for the CCA and faecal egg count over the three days of examination showed significant association with intensity of infection. Majority of the fluctuation (>85%) in *S. mansoni* test results by both methods were observed in children with light intensity of infection. Only 2 children with heavy intensity of infection by the six Kato-Katz method showed variation in faecal egg count across the three days, and none of the children with heavy intensity of infection showed changes in CCA test results over the three days of examination. As the Kato-Katz and the urine-CCA methods are less sensitive in light intensity infections [8,15], it is highly likely that large number of children could be miss-diagnosed in low endemic areas of the disease and in light intensity of infection. The fluctuation in *S. mansoni* infection results based on the Kato-Katz was also associated with area, where a higher change was observed in participants from Harbu area than those from Jiga area. This could be due to the large number of children with moderate (31.3%, 52/166) and heavy (8.4%, 14/166) infection from Harbu area than from the Jiga area (moderate = 16.0%, 26/163, heavy = 3.1%, 5/163). Other factors related with parasite strain could also be reasons for the difference in the magnitude of fluctuation observed in Harbu and Jiga areas. However, the fluctuation in both the CCA test scores and faecal egg count was not associated with age and sex.

The current finding supports the notion that level of *S. mansoni* CCA released in urine and amount of eggs excreted in stool could show day-to- day variation suggesting the necessity for several urine or stool samples collection on different days for more reliable diagnosis of *S. mansoni* infection using the urine-CCA cassette test or the Kato-Katz method. Based on the results of the current study, a minimum of three urine or stool samples collected on three different days might be important to diagnose *S. mansoni* infection using the urine-CCA cassette test or the Kato-Katz method with better accuracy. In addition, increasing the number of Kato-Katz slides prepared from each stool sample collected on a single day would be vital for reliable diagnosis of *S. mansoni* infection [7]. Although increasing the number of samples (preferably on different days) in both the Kato-Katz thick smear and the urine-CCA cassette test could increase the performance of both techniques for the diagnosis of *S. mansoni* infection [11-14], determining the maximum number of samples that can be used for estimating infection with a minimum level of miss-diagnosis of cases would be challenging based on the current study. However, the observation of considerable number of children who were positive on one of the three days, but negative on the other two days of examination or vice versa by both the CCA and Kato-Katz methods in the current study seems to suggest that at least three samples collected on three different days would be important for better evaluation of infection status of *S. mansoni* (e.g. for treatment purpose) by both techniques. Nevertheless, further studies with large number of samples collected on several days, considering both the cost benefit analysis and feasibility would be important for deciding the minimum number of samples required for accurate estimation of infection status. As collection of several samples from individuals on consecutive days would be more anesthetics for stool specimens, the urine-CCA cassette test that involves only urine collection seems less challenging and more feasible for diagnosing *S. mansoni* infection with increased sensitivity. On top of that, the current study showed a relatively less variation in urine-CCA results compared to Kato-Katz over the three days of examination. Thus, even a single urine-CCA cassette test would suffice for mapping and screening of *S. mansoni* infection at a reasonable cost.

## Conclusions

In conclusion, the current study showed day-to-day variation in CCA and Kato-Katz test results of children infected with *S. mansoni*. This day-to-day fluctuation in *S. mansoni* CCA test scores and egg counts demonstrates the necessity for more than one urine or stool samples to be collected on different days for more reliable diagnosis of *S. mansoni* infection. However, further studies might be important for estimating the minimum number of samples required in diagnosis of *S. mansoni* infection with better accuracy but with less challenges due to subject compliance and cost required.

## Competing interests

The authors declare that they have no competing interests.

## Authors’ contributions

AD & BE Conceived the idea. BE & TT collected the data. AD analyzed the data and drafted the manuscript. BE, GM & ML had intellectual contribution for the development of the manuscript. Finally all authors commented and approved the final manuscript.

## Pre-publication history

The pre-publication history for this paper can be accessed here:

http://www.biomedcentral.com/1471-2334/14/210/prepub
